# Casemix system for the elderly persons in Japan

**DOI:** 10.1186/1472-6963-12-S1-I7

**Published:** 2012-11-21

**Authors:** Jiro Okochi

**Affiliations:** 1 Tatsumanosato Geriatric Health Services Facilities, Osaka, Japan

## Background

Casemix classification systems for post acute care have been developed in several countries. The paradigm of each system is different depending on service and target population and the intension of the system itself. Japanese classification system is more suitable for long-term nursing care for elderly person. In contrast, RUGs system used in the US considers rehabilitation and medical service highly in their hierarchical system suitable as a sub-acute classification system.

## Development of Casemix system for sub-acute care

There are three important components when we are developing the Casemix system for sub-acute care.

1. Independent variables: such as patient functioning, disease category and aim of service use.

2. Outcome variables; such as work provided by health care Prof.essionals.

3. Incentives for facilities and client

These three components are interactively related. A weekly patients’ assessment in RUG assessment system gave nursing homes toward early discharge of the patient from Medicaid paid facilities. In contrast the nursing home tends to take patients with severe functional problem in Japanese system, regardless of their length of stay.

## Future of the sub-acute Casemix system with the ICF

The WHO has implemented the International Classification of Functioning, Disability and Health (ICF) in 2001. The ICF provides a broad description of functioning in the form of hierarchical categories and can be used as common taxonomy among health care Prof.essionals internationally. The adaptation of the ICF as common taxonomy to be used in sub-acute case mix system may solve problems of current sub-acute case mix systems. A Japanese experience of ICF adoptation in patient management will be discussed.

